# Effectiveness of a 3-Week Rehabilitation Program Combining Muscle Strengthening and Endurance Exercises Prior to Total Knee Arthroplasty: A Non-Randomized Controlled Trial

**DOI:** 10.3390/jcm12041523

**Published:** 2023-02-15

**Authors:** Takamasa Hashizaki, Yukihide Nishimura, Takahiro Ogawa, Chigusa Ohno, Ken Kouda, Yasunori Umemoto, Takaya Taniguchi, Hiroshi Yamada, Fumihiro Tajima

**Affiliations:** 1 Department of Rehabilitation Medicine, Wakayama Medical University, Wakayama 641-8509, Japan; 2 Department of Rehabilitation Medicine, Iwate Medical University, Morioka 020-8505, Japan; 3 Department of Rehabilitation Medicine, Aichi Medical University, 1-1 Yasagorimata, Nagakute 480-1195, Japan; 4 Department of Rehabilitation Medicine, Gifu City Hospital, Gifu 500-8513, Japan; 5 Department of Orthopedic Surgery, Naga Hospital, Wakayama 649-6414, Japan; 6 Department of Orthopedic Surgery, Wakayama Medical University, Wakayama 641-8509, Japan

**Keywords:** arthroplasty, knee joint, physical and rehabilitation medicine, physical fitness

## Abstract

We evaluated the effectiveness of a high-intensity preoperative resistance and endurance training program in improving physical function among patients scheduled for total knee arthroplasty. This non-randomized controlled trial included 33 knee osteoarthritis patients scheduled to undergo total knee arthroplasty at a tertiary public medical university hospital. Fourteen and nineteen patients were non-randomly assigned to intervention and control groups, respectively. All patients underwent total knee arthroplasty and a postoperative rehabilitation program. The intervention group participated in a preoperative rehabilitation program comprising high-intensity resistance and endurance training exercises to increase lower limb muscle strength and endurance capacity. The control group received only exercise instruction. The primary outcome was the 6-min walking distance, which was significantly higher in the intervention group (399 ± 59.8 m) than in the control group (348 ± 75.1 m) 3 months post-surgery. There were no significant differences between the groups 3 months post-surgery in muscle strength, visual analog scale, WOMAC-Pain, range of motion of knee flexion, and extension. A 3-week preoperative rehabilitation program combining muscle strengthening and endurance training improved endurance 3 months after total knee arthroplasty. Thus, preoperative rehabilitation is important for improving postoperative activity.

## 1. Introduction

Knee osteoarthritis is the most common joint disease worldwide, with significant personal and societal impacts on musculoskeletal pain, disability, and socioeconomic costs [1,2]. Patients with knee osteoarthritis are limited in their activities of daily living because of pain, decreased muscle strength, and endurance capacity [3,4,5,6]. Total knee arthroplasty is widely used to treat advanced knee osteoarthritis, as it was shown to reduce pain and facilitate a gradual return to activities of daily living among patients [7,8].

Nevertheless, studies have shown that physical function declines after total knee arthroplasty for knee osteoarthritis, which requires long periods of standard physical therapy for improvement. The quadriceps’ muscle strength temporarily decreases after surgery and takes 3 months to recover to preoperative levels; approximately 1 year is required for the quadriceps’ muscle strength on the operated side to recover to the same level as that on the healthy side [9]. In terms of endurance after total knee arthroplasty, studies have shown that 6-min walking distance, an indicator of walking ability and cardiopulmonary function, is reduced by approximately 39% at 1 month after surgery, even with standard postoperative physical therapy [10]. A period of 1.5–3 months is required for a patient’s 6-min walking distance to return to preoperative levels [11,12].

Numerous studies investigated the effectiveness of preoperative training programs in improving physical function after total knee arthroplasty [13,14,15,16,17]. Calatayud et al. [16] reported that 8 weeks of muscle strength training before total knee arthroplasty improved postoperative knee extensor strength, pain, and walking ability. In contrast, a meta-analysis found that preoperative muscle strength training did not improve postoperative physical function [18]; however, none of the included studies evaluated whether preoperative training could improve endurance capacity. Therefore, a combination of preoperative muscle strengthening and endurance training may be potentially effective in improving postoperative endurance.

Three weeks of preoperative endurance exercise training for patients with myocardial infarction and chronic pulmonary obstructive disease can reportedly improve maximal oxygen consumption and the 6-min walking distance, which are indicators of endurance [19,20]. Therefore, it is estimated that at least 3 weeks of endurance exercise training is necessary to improve endurance in patients before total knee arthroplasty. We hypothesized that a 3-week preoperative training program combining muscle strengthening exercises with endurance exercises would improve postoperative endurance in patients after total knee arthroplasty. This study evaluates whether 3 weeks of muscle strengthening and endurance training in preoperative TKA patients improves knee extensor strength and six-minute walking distance preoperatively and promotes recovery of knee extensor strength and six-minute walking distance after 3 months post-operation compared to non-preoperative exercise therapy patients.

## 2. Materials and Methods

### 2.1. Study Setting and Design

This non-randomized controlled trial was conducted at the Department of Rehabilitation Medicine and Orthopedic Surgery, University Hospital, between 21 May 2018 and 31 August 2020. This study was conducted in compliance with the Declaration of Helsinki, approved by the Institutional Review Board of Wakayama Medical University (no. 2309), and registered in the University Hospital Medical Information Network (ID.UMIN 000032568).

### 2.2. Study Participants

All data were collected at Wakayama Medical University. Patients were included if they were >50 years old, diagnosed with advanced idiopathic knee osteoarthritis according to the radiological criteria of the American College of Rheumatology Guidelines, and scheduled for unilateral or bilateral total knee arthroplasty in our hospital. Patients were excluded if they had active or recently treated infections, were unable to walk independently or with supervision for 6 min, or had any medical conditions for which exercise was contraindicated. All patients provided written informed consent before study commencement.

### 2.3. Surgical Procedures and Postoperative Rehabilitation

All patients underwent total knee arthroplasty, which was performed by three experienced orthopedic surgeons using the same standardized surgical technique. In all cases, the posterior cruciate ligament was removed, and the operations were performed using a tourniquet. As part of usual care, all patients received the same in-hospital postoperative rehabilitation protocol after total knee arthroplasty. Patients entered the rehabilitation program within 24–48 h after surgery. Patients were mobilized out of bed in the initial stages of rehabilitation. All out-of-bed activities were performed under the supervision of a physical therapist. Rehabilitation usually involved resistance exercises, stretching exercises, activities of daily living exercises, aerobic training, and gait training. The patients performed the rehabilitation exercises twice daily (from Monday to Saturday) during their hospital stay; each session lasted 1 h.

### 2.4. Intervention

The 3-week preoperative training program, combining muscle strengthening exercises with endurance exercise, was created by modifying an exercise program from a previous study that was effective in restoring knee extensor strength 3 months after surgery as a preoperative exercise therapy for TKA [16], and it was conducted by physicians and physiotherapists at the Department of Rehabilitation Medicine, Wakayama Medical University. The intervention group participated in 90-min training sessions three times per week, starting 3 weeks before surgery. The rehabilitation program was designed to increase lower limb muscle strength and endurance capacity. A physical therapist supervised all training sessions.

Each training session started with body weight exercises consisting of five sets of 20 step-up and calf-raise repetitions and 10 sets of 10 repetitions of wall squats. After body weight exercises, patients performed four sets of a 30-s double-leg stance and four sets of a 15-s single-leg stance on an unstable device. Resistance training consisted of three different exercises: knee extension, hip extension, and hip abduction. Additionally, leg presses were performed using a machine (SELECTION700, TechnoGym, Tokyo, Japan). Training intensity was based on the patient’s ability to execute a 10-repetition maximum. Patients performed five sets of 10 repetitions for each resistance exercise. The hand (818E, Monark, Vansbro, Sweden) and leg cycle ergometer (915E, Monark, Vansbro, Sweden) exercises were performed at 60% of the heart rate reserve for 20 min. The Karvonen formula ((220 − age − resting heart rate) × % intensity + resting heart rate) was used to determine the heart rate that had to be maintained to achieve the desired and prescribed exercise intensity. The training session ended with light static stretching of the hip abductors, hip flexors, hip extensors, knee extensors, knee flexors, and ankle plantar flexors. Patients consumed 10 g of protein in a jelly drink immediately after each training session. No activities were implemented to increase compliance or adherence. The control group received only exercise instruction, including quadricep strength and landing, at the time of outcome assessment one month prior to surgery.

### 2.5. Measures

The following outcomes were evaluated: The Western Ontario and McMaster Universities Osteoarthritis Index-Pain (WOMAC-Pain), isometric strength (knee extension) test, passive knee range of motion (flexion and extension) test, 6-min walking test, and visual analog scale (VAS) for pain. The primary outcome was the 6-min walking distance, whereas the other evaluations were secondary outcomes. Each outcome was evaluated by skilled physical therapists. Outcomes were assessed at (1) baseline (3 weeks before surgery), (2) after 3 weeks of preoperative training, (3) 1 month after total knee arthroplasty, and (4) 3 months after total knee arthroplasty.

#### 2.5.1. Western Ontario and McMaster Universities Osteoarthritis Index (WOMAC)

The WOMAC is a 24-item, self-administered questionnaire that includes subscales for pain (5 items), joint stiffness (2 items), and physical function (17 items) [10]. It is rated on a 5-point Likert scale (0–4), with higher scores indicating lower symptom or disability levels. The questionnaire is scored by summating each subscale or by computing a global score. In this study, we measured only five items of pain subscale.

#### 2.5.2. Isometric Strength Test

The isometric strength of knee extensors was measured using a portable handheld dynamometer (μTas F-1; Anima, Tokyo, Japan). Patients were positioned in a seated position (with 90° hip flexion and knee flexion) during the measurement of maximal isometric knee extension strength. Patients were instructed to remain seated in an upright position and to place both hands on their upper legs to avoid compensation. The ankle pad was placed 3 cm proximal to the medial malleolus. Patients were instructed to contract their quadriceps as forcefully as possible, thus pushing their legs against the pad. The dynamometer was positioned perpendicular to the tibia and fixated by a belt to the plinth. The distance from the dynamometer to the knee joint center was measured. All patients performed two isometric maximal voluntary contractions, and the higher score was selected for further analysis. Peak torque values were calculated via the formula, (peak score) × 9.80665 × (distance from the dynamometer to the knee joint center); they were normalized to body weight and reported as Nm/kg. Dynamometry is considered the gold standard of muscle strength assessment, and dynamometry tests of knee extensor muscles in patients with knee osteoarthritis have been previously demonstrated to be reliable [12,13].

#### 2.5.3. Passive Knee Range of Motion Test

Passive range of motion of knee joint flexion and extension of the affected knee was measured by goniometry. With the patient in the supine position, the fulcrum of the goniometer was placed over the lateral epicondyle, with one 30-cm arm pointed toward the major trochanter of the femur and the other pointed toward the lateral malleolus. This method has been shown to be reliable and valid in patients with a restricted knee range of motion [14].

#### 2.5.4. Six-Minute Walking Distance Test

The 6-min walking test measures patients’ maximum walking distance in 6 min. Patients were instructed to walk as far as possible in 6 min in a safe manner in an undisturbed 30-m corridor [16,17], and they were required to walk from one end to the other and then turn around. The use of assistive devices was allowed if necessary [16]. Values were recorded to the nearest centimeter.

#### 2.5.5. Visual Analog Scale

In this assessment, the patient was shown a 10-cm straight line with the left end marked as “no pain” and right end marked as “the greatest pain imaginable.” The patient was then asked to indicate where their current pain corresponded to on the line, and then the distance was measured from the left end of the line to the mark made by the patient. This measurement corresponded to pain intensity (range 0.0–10.0 cm).

### 2.6. Statistical Analysis

The planned sample size of 50 was calculated using JMP Pro (JMP, USA) and based on the following parameters: dropout rate of 0.3, power of 0.8, significance level of 0.05, and minimally detectable difference of 1 standard deviation (SD) between the groups.

Differences in age, height, weight, body mass index, and outcomes (WOMAC, isometric strength of the knee extensors, passive knee range of motion, 6-min walking test) between the groups were evaluated with the independent *t*-test. Intragroup differences in outcomes were assessed using one-way analysis of variance (Friedman test). Post-hoc analysis for items with significant differences was performed using Dunn’s multiple comparison test. Data are expressed as mean ± SD. The level of statistical significance was set at *p* < 0.05. All statistical analyses were conducted using Graph Pad Prism 7 (GraphPad Software Inc., San Diego, CA, USA).

## 3. Results

**Figure 1:**
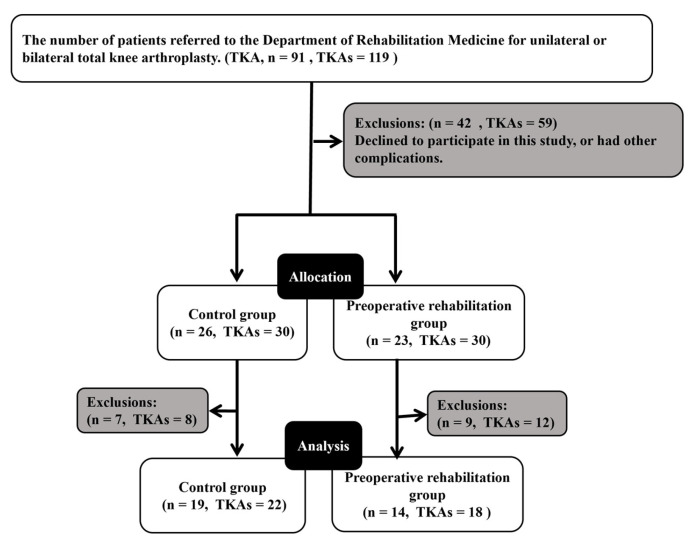
Flow diagram of patient selection and progress through the phases of the study. TKA, total knee arthroplasty.

Ninety-one patients (119 knees) who provided consent between May 2018 and August 2020 were assessed by an orthopedic surgeon and included in the study. Among these, 42 patients (59 knees) were excluded because they declined to participate in this study or had other complications (Figure 1). Thus, forty-nine patients (60 knees) were assigned based on their ability to commute to the hospital: 23 (30 knees) were allocated to the intervention group (in-hospital preoperative exercise training) and 26 (30 knees) were non-randomly allocated to the control group (outpatient preoperative exercise training). Age and sex were matched to minimize potential bias caused by non-randomization. Nine and seven patients in the intervention and control groups, respectively, withdrew from the study after group allocation. Some patients in the control group withdrew owing to difficulties in commuting to the hospital because of distance (*n* = 4), postponement of surgery (*n* = 1), difficulty in scheduling preoperative assessments (*n* = 1), and intraoperative complications (*n* = 1). Some patients in the intervention group withdrew owing to the following reasons: history of previous knee surgery (*n* = 1), postponement of surgery (*n* = 3), intraoperative complications (*n* = 1), postoperative complications (*n* = 2), surgery for another disease during the study period (*n* = 1), and physical illness during the intervention period (*n* = 1). No patients in the intervention group dropped out because of increased pain or fatigue caused by the training exercises. Thus, 14 patients (18 knees) in the intervention group and 19 (22 knees) in the control group were included in the final analysis. Patients could not be blinded because of the nature of the preoperative exercise program. The evaluator was blinded via their location in a separate room from the implementer of the intervention.

Patient characteristics are summarized in Table 1; no significant differences were found between the groups. There were no adverse events due to the intervention throughout the study period.

**Figure 2:**
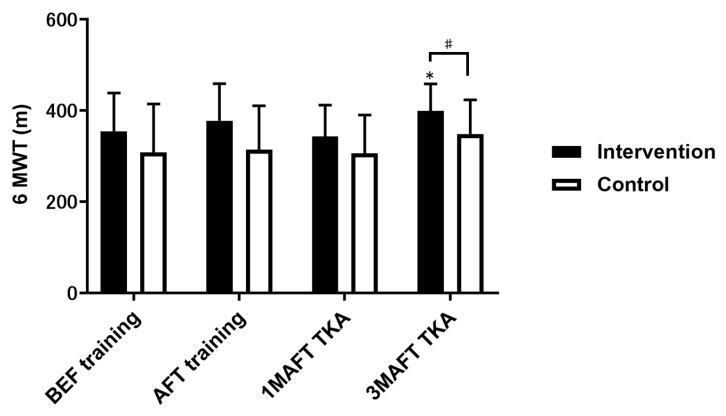
Comparison of 6MWT results between the intervention and control groups. Data are presented as mean ± standard deviation. * *p* < 0.05, compared with BEF training. # *p* < 0.05, compared with the control group. 6MWT, 6-min walking test; BEF, baseline 3 weeks before surgery; AFT, after 3 weeks of preoperative training; M, month; TKA, total knee arthroplasty.

The 6-min walking distance in the intervention group was significantly greater 3 months after surgery than at baseline; no significant change was observed in the control group. Significantly higher values were observed in the intervention group than in the control group 3 months after total knee arthroplasty (Figure 2).

**Figure 3:**
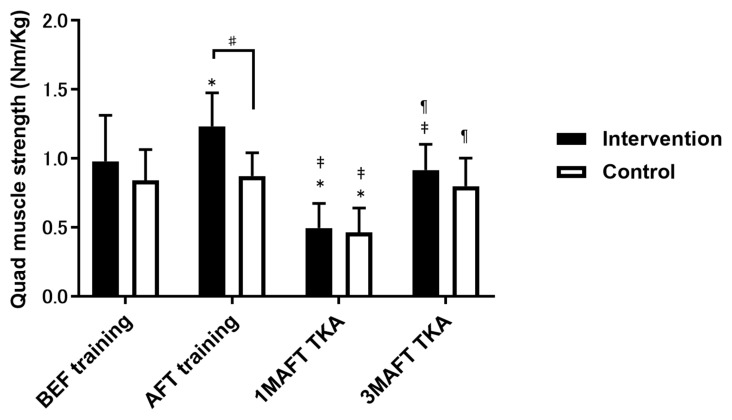
Comparison of quadriceps muscle strength between the intervention and control groups. Data are presented as mean ± standard deviation. *, *p* < 0.05, compared with BEF training; ‡, *p* < 0.05, compared with AFT training; ¶, *p* < 0.05, compared with 1M AFT TKA; #, *p* < 0.05, compared with the control group. BEF, baseline 3 weeks before surgery; AFT, after 3 weeks of preoperative training; M, month; TKA, total knee arthroplasty. Data are presented as mean ± standard deviation.

Quadricep muscle strength (on the operative side) in the intervention group significantly improved after 3 weeks of preoperative training compared with that at baseline; however, a significant decrease was observed 1 month after total knee arthroplasty. The control group did not exhibit a significant difference in quadricep muscle strength before surgery compared to that at baseline; however, a significant decrease was observed 1 month after surgery. In either group, the quadriceps’ muscle strength (on the non-operative side) did not change over time. Although there were no significant differences between the groups after 3 weeks of preoperative training, the intervention group had significantly higher values at baseline and at 1 and 3 months postoperatively (Figure 3).

The range of motion for knee extension significantly improved in both groups at 1 and 3 months after total knee arthroplasty compared to their performance at baseline and after 3 weeks of preoperative training. The range of motion for knee flexion significantly decreased in the intervention group 1 month after total knee arthroplasty compared to their performance after 3 weeks of preoperative training. The control group did not differ significantly at each time period. There were no significant differences in the range of motion for knee flexion or extension between the groups (Table 2). VAS score (operative side) significantly improved in both groups at 1 and 3 months after total knee arthroplasty compared with that at baseline and after 3 weeks of preoperative training. There were no significant differences between the groups at any time (Table 2).

WOMAC-Pain scores on the operative side significantly improved in the intervention group 3 months after total knee arthroplasty compared with those at baseline and after 3 weeks of preoperative training. The control group exhibited significant improvements at 1 and 3 months postoperatively compared to their performance at baseline and after 3 weeks of preoperative training. Between-group comparisons indicated that the intervention group had significantly lower scores than the control group at baseline (Table 2).

## 4. Discussion

This non-randomized controlled study evaluated the effectiveness of a 3-week preoperative rehabilitation program in improving physical function among patients scheduled to undergo total knee arthroplasty. We found that preoperative exercise training resulted in a significantly greater 6-min walking distance relative to the control group 3 months after surgery. In the intervention group, knee extension strength of the affected leg increased significantly compared to that of the control group, but 3 months after surgery, as in the control group, it decreased to the same level as 3 weeks before total knee arthroplasty. Pain in both groups improved at 3 months postoperatively, but there was no difference between the groups, and exercise therapy in the month before surgery had no effect on postoperative pain improvement.

The 6-min walking test is frequently used for assessing cardiopulmonary function, and its results are known to be correlated with maximal oxygen uptake [21], muscle strength [22], and arthritis [23]. A previous study reported that 4 weeks of muscle strength training alone did not improve the 6-min walking distance, despite increasing muscle strength [17]. In contrast, other studies have reported a correlation between preoperative lower limb muscle strength on the operative side and postoperative 6-min walking distance [24]. The 3-week preoperative exercise training in the present study was aimed at improving cardiopulmonary function via an increase in both muscle strength and endurance; these effects, in conjunction with reduction in the severity of arthritis and pain after total knee arthroplasty, may have contributed to the improvement of the 6-min walking distance 3 months postoperatively. The novelty of this study is that combination exercise during the 3 weeks before surgery improved cardiopulmonary function during the 3 months after surgery. The reason for the lack of improvement at 1 month postoperatively was residual postoperative muscle weakness.

Muscle hypertrophy occurs 6–7 weeks after the start of muscle strength training, and muscle strength gains before that time are mainly due to neural adaptations [25,26]. A study that evaluated an 8-week preoperative muscle strength training program in patients scheduled to undergo total knee arthroplasty found that the muscle strength gains obtained preoperatively were maintained postoperatively [16]. In the present study, the duration of preoperative muscle strength training was only 3 weeks, and the muscle strength decreased to the same level as that of the control group at 1 and 3 months postoperatively, suggesting that muscle strength gains in the intervention group were due to neural adaptation. Thus, the duration of preoperative exercise training is an important factor for sustained muscle strength gain after total knee arthroplasty.

Preoperative exercise training in the present study was not effective in reducing postoperative pain. Previous studies reported a reduction in pain with 30–60 min of training sessions (three sessions per week for 24 weeks) comprising 2–3 sets of 8–12 repetitions of resistance exercises [27]. As we provided an exercise training program of similar intensity and frequency in the present study, the short program duration of 3 weeks was likely the main factor related to the lack of effect on postoperative pain.

This study included a mix of unilateral and bilateral TKA patients. Therefore, differences between unilateral and bilateral TKA may affect physical function at 3 months postoperatively. However, because this study was designed with a combined sample size of unilateral and bilateral TKA, it is difficult to examine differences between unilateral and bilateral TKA, and because there is no significant difference in the proportion of bilateral TKA patients in the Control and Intervention groups, it is likely that surgery did not influence the results of this study. The difference in the percentage of bilateral TKA patients in the Control and Intervention groups was insignificant.

In this study, the exercise program combined endurance training with short-term preoperative rehabilitation, which was effective in improving endurance 3 months after total knee arthroplasty. Walking ability may also be improved in patients with osteoarthritis of other lower extremity joints (e.g., hip joints) through similar preoperative rehabilitation programs. However, further studies with preoperative exercise training programs of longer duration are required to analyze potential effects on postoperative pain.

### Study Limitations

Our study has some limitations. The main limitation was the difficulty in achieving a completely random assignment of patients to the intervention and control groups. This was attributed to the geographical location of the study site, which was often difficult to access for patients with knee osteoarthritis.

Another limitation was that this type of combination exercise program has multiple effects on fitness. Thus, it is unclear which contributed more to the postoperative outcomes because anaerobic endurance was integrated with aerobic training.

## 5. Conclusions

This study determined the effects of a 3-week preoperative rehabilitation program combining high-intensity resistance exercise and endurance training on physical function in patients scheduled to undergo total knee arthroplasty. The results show that this program improved endurance 3 months after surgery.

## Figures and Tables

**Table 1:** Demographic characteristics of the patients.

	Control	Intervention	95% Confidence Interval	*p*-Value
Number of patients	19	14		
Age (years)	77.7 ± 6.9	73.7 ± 4.7	−0.342 to 0.387	0.07
Height (cm)	153.1 ± 7.7	155.0 ± 10.3	−0.083 to 0.043	0.52
Weight (kg)	56.5 ± 9.0	60.3 ± 9.6	−10.45 to 2.905	0.25
Body mass index (kg/m^2^)	22.8 ± 6.3	25.1 ± 3.2	−6.083 to 1.491	0.23
Sex (female/male)	3/16	5/9		
Number of bilateral TKA	4	3	-	0.37
Length of stay (days)	27.4 ± 16	21.5 ± 3.8	−3.452 to 15.29	0.2

**Table 2:** Comparison of outcome measures between the intervention and control groups.

Variable	Testing Time	Control (*n* = 22 TKAs)	Intervention (*n* = 18 TKAs)	95% Confidence Interval	*p*-Value
ROM flexion (°)	BEF training	120.1 ± 14.3	123.0 ± 14.4	−12.09 to 0.364	NS
	AFT training	121.0 ± 14.7	127.7 ± 10.9	−15.18 to 1.76	NS
	1-month AFT TKA	118.9 ± 8.3	121.8 ± 12.0 **	−9.383 to 3.645	NS
	3-month AFT TKA	121.6 ± 11.5	124.0 ± 10.7	−9.534 to 4.806	NS
ROM extension (°)	BEF training	−10.8 ± 5.5	−11.1 ± 6.5	−3.545 to 4.11	NS
	AFT training	−11.1 ± 5.1	−8.3 ± 5.5	−6.178 to 0.663	NS
	1-month AFT TKA	−5.3 ± 3.9 *^,^**	−4.2 ± 2.2 *^,^**	−3.209 to 1.017	NS
	3-month AFT TKA	−4.5 ± 4.4 *^,^**	−4.2 ± 3.4 *^,^**	−2.848 to 2.293	NS
VAS (cm)	BEF training	6.7 ± 2.2	5.5 ± 2.3	−0.25 to 2.611	NS
	AFT training	7.3 ± 2.3	5.9 ± 2.4	−0.147 to 2.867	NS
	1-month AFT TKA	2.4 ± 2.4 *^,^**	2.3 ± 1.9 *^,^**	−1.333 to 1.501	NS
	3-month AFT TKA	1.3 ± 1.3 *^,^**	1.2 ± 1.2 *^,^**	−0.766 to 0.903	NS
WOMAC pain (m)	BEF training	10.2 ± 3.0	8.3 ± 2.7	0.102 to 3.797	0.039
	AFT training	10.2 ± 2.9	9.2 ± 3.3	−0.967 to 2.977	NS
	1-month AFT TKA	5.8 ± 4.3	6.0 ± 1.9 *^,^**	−2.404 to 2.04	NS
	3-month AFT TKA	3.5 ± 3.0 *^,^**	4.2 ± 2.4 *^,^**	−2.392 to 1.15	NS

Data are presented as mean ± standard deviation. *, *p* < 0.05, compared with BEF training; **, *p* < 0.05, compared with AFT training. BEF, baseline 3 weeks before surgery; AFT, after 3 weeks of preoperative training; NS, not significant; ROM, range of motion; TKA, total knee arthroplasty; VAS, visual analog scale; WOMAC, Western Ontario and McMaster Universities Osteoarthritis Index.

## Data Availability

The datasets generated and/or analyzed during the current study are available from the corresponding author on reasonable request. The data are not publicly available owing to privacy reasons.

## References

[1] Glyn-Jones S., Palmer A.J.R., Agricola R., Price A.J., Vincent T.L., Weinans H., Carr A.J. (2015). Osteoarthritis. Lancet.

[2] Allen K.D., Golightly Y.M. (2015). State of the evidence. Curr. Opin. Rheumatol..

[3] Jones C.A., Voaklander D.C., Johnston D.W., Suarez-Almazor M.E. (2000). Health related quality of life outcomes after total hip and knee arthroplasties in a community based population. J. Rheumatol..

[4] Berman A.T., Bosacco S.J., Israelite C. (1991). Evaluation of total knee arthroplasty using isokinetic testing. Clin. Orthop. Relat. Res..

[5] Petterson S.C., Barrance P., Buchanan T., Binder-Macleod S., Snyder-Mackler L. (2008). Mechanisms underlying quadriceps weakness in knee osteoarthritis. Med. Sci. Sports Exerc..

[6] Ko V., Naylor J.M., Harris I.A., Crosbie J., Yeo A.E. (2013). The six-minute walk test is an excellent predictor of functional ambulation after total knee arthroplasty. BMC Musculoskelet. Disord..

[7] Carr A.J., Robertsson O., Graves S., Price A.J., Arden N.K., Judge A., Beard D.J. (2012). Knee replacement. Lancet.

[8] Nilsdotter A.K., Toksvig-Larsen S., Roos E.M. (2009). Knee arthroplasty: Are patients’ expectations fulfilled? A prospective study of pain and function in 102 patients with 5-year follow-up. Acta Orthop..

[9] Schroer W.C., Diesfeld P.J., Reedy M.E., LeMarr A.R. (2010). Isokinetic strength testing of minimally invasive total knee arthroplasty recovery. J. Arthroplasty.

[10] Bade M.J., Kohrt W.M., Stevens-Lapsley J.E. (2010). Outcomes before and after total knee arthroplasty compared to healthy adults. J. Orthop. Sports Phys. Ther..

[11] Bruun-Olsen V., Heiberg K.E., Wahl A.K., Mengshoel A.M. (2013). The immediate and long-term effects of a walking-skill program compared to usual physiotherapy care in patients who have undergone total knee arthroplasty (TKA): A randomized controlled trial. Disabil. Rehabil..

[12] Kittelson A., Carmichael J., Stevens-Lapsley J., Bade M. (2022). Psychometric properties of the 4-meter walk test after total knee arthroplasty. Disabil. Rehabil..

[13] Silkman Baker C., McKeon J.M. (2012). Does preoperative rehabilitation improve patient-based outcomes in persons who have undergone total knee arthroplasty? A systematic review. PMR.

[14] Kwok I.H., Paton B., Haddad F.S. (2015). Does preoperative physiotherapy improve outcomes in primary total knee arthroplasty? -A systematic review. J. Arthroplasty.

[15] Chesham R.A., Shanmugam S. (2017). Does preoperative physiotherapy improve postoperative, patient-based outcomes in older adults who have undergone total knee arthroplasty? A systematic review. Physiother. Theory Pract..

[16] Calatayud J., Casaña J., Ezzatvar Y., Jakobsen M.D., Sundstrup E., Andersen L.L. (2017). High-intensity preoperative training improves physical and functional recovery in the early post-operative periods after total knee arthroplasty: A randomized controlled trial. Knee Surg. Sport. Traumatol. Arthrosc..

[17] Skoffer B., Maribo T., Mechlenburg I., Hansen P.M., Søballe K., Dalgas U. (2016). Efficacy of preoperative progressive resistance training on postoperative outcomes in patients undergoing total knee arthroplasty. Arthritis Care Res..

[18] Chen H., Li S., Ruan T., Liu L., Fang L. (2018). Is it necessary to perform prehabilitation exercise for patients undergoing total knee arthroplasty: Meta-analysis of randomized controlled trials. Phys. Sportsmed..

[19] Andjic M., Spiroski D., Ilic Stojanovic O., Vidakovic T., Lazovic M., Babic D., Ristic A., Mazic S., Zdravkovic M., Otasevic P. (2016). Effect of short-term exercise training in patients following acute myocardial infarction treated with primary percutaneous coronary intervention. Eur. J. Phys. Rehabil. Med..

[20] von Leupoldt A., Hahn E., Taube K., Schubert-Heukeshoven S., Magnussen H., Dahme B. (2008). Effects of 3-week outpatient pulmonary rehabilitation on exercise capacity, dyspnea, and quality of life in COPD. Lung.

[21] Zhang Q., Lu H., Pan S., Lin Y., Zhou K., Wang L. (2017). 6MWT performance and its correlations with VO_2_ and handgrip strength in home-dwelling mid-aged and older Chinese. Int. J. Environ. Res. Public Health.

[22] Enright P.L., McBurnie M.A., Bittner V., Tracy R.P., McNamara R., Arnold A., Newman A.B., Cardiovascular Health Study (2003). The 6-min walk test: A quick measure of functional status in elderly adults. Chest.

[23] Enright P.L. (2003). The six-minute walk test. Respir. Care.

[24] van Leeuwen D.M., de Ruiter C.J., Nolte P.A., de Haan A. (2014). Preoperative strength training for elderly patients awaiting total knee arthroplasty. Rehabil. Res. Pr..

[25] Phillips S.M. (2000). Short-term training: When do repeated bouts of resistance exercise become training?. Can. J. Appl. Physiol..

[26] Kraemer W.J., Fleck S.J., Evans W.J. (1996). Strength and power training: Physiological mechanisms of adaptation. Exerc. Sport Sci. Rev..

[27] Turner M.N., Hernandez D.O., Cade W., Emerson C.P., Reynolds J.M., Best T.M. (2020). The role of resistance training dosing on pain and physical function in individuals with knee osteoarthritis: A systematic review. Sport. Health.

